# Usefulness of a New Effective Dose Conversion Factor Using Size-Specific Dose Estimate (SSDE) in Abdominopelvic Region

**DOI:** 10.7759/cureus.87499

**Published:** 2025-07-08

**Authors:** Masato Takanashi, Yoshiaki Katada, Isao Kuroda, Masataka Hoshina, Masayoshi Matsushita, Koichi Masuda, Shinji Sugahara

**Affiliations:** 1 Department of Radiology and Radiation Oncology, Tokyo Medical University Ibaraki Medical Center, Inashiki-gun, JPN; 2 Department of Urology, Tokyo Medical University Ibaraki Medical Center, Inashiki-gun, JPN

**Keywords:** compute tomography, dose length product, dose management software, effective dose, radiation protection, size-specific dose estimates

## Abstract

The purpose of this study was to determine a new conversion factor (k_SSDE-LP_) that would allow more accurate calculation of the effective dose that is minimally influenced by patient body size, using the Dose Management Software (DMS), and to verify its accuracy in estimating patient-specific effective dose compared to existing methods. A simple regression equation was obtained using the product of the size-specific dose estimate (SSDE) and scan length on the horizontal axis and the effective dose on the vertical axis, and the slope was taken as the effective dose conversion coefficient, k_SSDE-LP_. Similarly, the slope obtained from another simple regression equation, using the dose-length product (DLP) on the horizontal axis and effective dose on the vertical axis, was defined as k_DLP_. The effective dose conversion factors and coefficients of determination (R^2^) were compared for males, females, and both sexes. The DLP, SSDE, scan length, and effective dose, which are dose indices necessary for determining effective dose conversion coefficients, were obtained from the DMS. The k_SSDE-LP_ values in males, females, and both sexes were 0.012 (R^2^ = 0.997), 0.014 (R^2^ = 0.996), and 0.013 (R^2^ = 0.993), respectively. Using the SSDE, a dose index that takes into account information about the patient's physique, we calculated the k_SSDE-LP_, which reflects the current tissue weighting coefficients and mathematical voxel phantom and does not easily deviate from the regression equation even in the high-weight group.

## Introduction

In Japan, the legal framework for the safe use of medical radiation has become more stringent, with the Ministerial Ordinance partially amending the Ordinance for Enforcement of the Medical Service Act, and the number of items that medical institutions providing radiological treatments must comply with has increased significantly. In this context, effective dose is a useful indicator for estimating the risk of stochastic effects. To calculate the effective dose, it is first necessary to determine the absorbed dose in each organ, either by measurement or by estimation. Then, radiation weighting factors and tissue weighting factors are multiplied and integrated. One method for estimating the absorbed doses by organs is to use Monte Carlo simulations. However, this requires specialized software, such as the Dose Management Software (DMS) or stand-alone programs, such as the NCI dosimetry system for computed tomography (NCICT). The International Commission on Radiological Protection (ICRP) Publication 102, published by the ICRP, provides k_Conventional_ (mSv・mGy-1・cm-1) (defined as k_Conventional_ in this study to distinguish it from other k values), which can be multiplied by the dose-length product (DLP) to obtain the effective dose and has been used in various studies. However, k_Conventional_ has several problems [1]. First, the tissue weighting factor is derived from ICRP Publication 60 rather than from the latest ICRP Publication 103, and second, the mathematical phantom adopted is outdated, so that the latest epidemiological findings and techniques have not been incorporated, resulting in frequent deviations from the values that reflect the latest findings [2-4].

The size-specific dose estimate (SSDE) proposed by the American Association of Physicists in Medicine (AAPM) in AAPM 204 and 220 takes into account the patient’s body size as compared to the CTDI_vol_ used in ICRP Publication 135 and Diagnostic Reference Levels (DRLs) 2020 [5-9]. Japanese people of standard physique are smaller than that reflected by the reference phantom diameter (32 cm), and CTDI_vol_, which is simply an instrument output value, often provides an underestimation when used for estimating patient doses [10]. Therefore, the SSDE, which provides a dose correction according to body size, is expected to be utilized in the future. While DLP is a product of CTDI_vol_ and scan length, we hypothesized that the SSDE-length product (in this study, SSDE-LP is defined as the product of SSDE and scan length), where CTDI_vol_ is replaced by SSDE, would allow a more accurate estimation of the effective dose. In a previous study using SSDE-LP, Martin et al. multiplied SSDE-LP by a correction factor for scan length based on the body size to obtain a regression equation that deviates less than 5% from the estimate obtained using NCICT, a dose estimation software [11]. In addition, as Martin et al. have moved toward optimizing medical exposures for individual patients, there is a growing demand for more specific risk-related quantities that take into account more detailed dosimetric information [11]. Thus, they state that patient-specific effective doses provide a better assessment of the doses for individual patients and are a step toward the development of risk-relevant quantities for individual patients, demonstrating the close relationship between the SSDE and effective dose and suggesting that simple formulas can be used to determine patient-specific effective doses. However, it has been reported that estimates of the effective dose obtained with DMS used for medical exposure estimation vary greatly depending on the product [12]. In addition, the characteristics of the automatic tube current adjustment mechanism in computed tomography (CT) machines vary by the manufacturer and among machines, so that the X-ray output is not always the same even when the same patient is imaged [13-14]. Although the regression equations derived from previous studies are informative, it is necessary to understand the regression equations derived from the combination of the CT equipment and DMS used at the facility in question in order to use them to explain the results to the patients at the facility. To the best of our knowledge, there is no report of a similar study using Aquilion ONE/PRISM Edition (TSX-306A: Canon Medical Systems Inc.), the CT equipment used at our hospital, and Radimetrics (Radimetrics ver3.3, Bayer Medical Care Inc., USA), the DMS used at our hospital. The purpose of this study was to derive a new conversion factor that would be less influenced by the body size than k_Conventional_ and would allow more accurate calculation of the effective dose utilizing DMS, and we derived the conversion factor k_SSDE-LP_ (defined as k_SSDE-LP_ in this study). We also verified whether k_SSDE-LP_ might be more useful in terms of the estimation accuracy as compared with k_Conventional_ and k_DLP_.

This could improve patient-specific risk assessment and communication in clinical practice.

## Materials and methods

SSDE can be calculated from the effective diameter (ED) proposed in AAPM 204 or from the water equivalent diameter (WED) proposed in AAPM 220; we calculated it using WED. As shown in the formula, WED includes CT values in the numerator, and therefore, arm hanging positions and metal implants were excluded from the specimens, as they could result in overestimation of the body size. Our calculation methods for ED and WED are described below.

Effective diameter = $\sqrt{AP\times LAT}$

where $AP$ denotes anteroposterior axial image,

and $LAT$ denotes lateral axial image.

Water Equivalent Diameter = $2\sqrt{[\frac{1}{1000}\overline{CT(x,y)}_{ROI}+1]\frac{A_{ROI}}{\pi}}$

where $\overline{CT(x,y)}_{ROI}$ denotes the average CT value in the Region Of Interest (ROI),

and $A_{ROI}$ denotes the total area in the ROI.

A total of 670 consecutive patients underwent abdominopelvic CT examinations between 2020 and 2021 at our hospital. Of these, the data of 377 cases from 2020 were used to derive the regression equation in this study, and the data of 293 cases from 2021 were used to verify its accuracy (Table 1). We use the TSX-306A CT equipment at our hospital. Microsoft Office Excel 2019 (Microsoft, Redmond, WA) was used for the analyses. Specifically, we derived the linear regression equation for the regression model using Microsoft Office Excel.

**Table 1:** Number of samples and age statistics in the years 2020 and 2021

		Male	Female
2020	Number of sample (N)	224	153
	Age (mean ± SD)	63 ± 18	60 ± 21
2021	Number of sample (N)	159	134
	Age (mean ± SD)	64 ± 17	63 ± 19

The protocol used for abdominopelvic CT examinations at our hospital is described below (Table 2). Scanning method, helical scanning; tube voltage, 120 kV. Volume exposure control (EC) is used as the automatic exposure control mechanism; Volume EC is a 3D mA modulation that automatically modulates the tube current in the XYZ direction. Rotation time, 500 msec; Pitch Factor, 0.813. A nominal single collimation width of 0.5 mm is used. Detector rows, 80; setting standard deviation, 15.

**Table 2:** CT examination protocol used in this study

Scan Type	Helical
Tube Voltage (kV)	120
Volume EC (XYZ Tube Current Modulation)	ON
Rotation Time (msec)	500
Pitch Factor	0.813
Nominal Single Collimation Width (mm)	0.5
Number of Detector Rows	80
Nominal Total Collimation Width (mm)	40
Setting Standard Deviation	15

The horizontal axis was set to SSDE-LP, which was calculated by multiplying the SSDE calculated from the axial images by the scan length using Radimetrics, which is the system used at our hospital. Similarly, the vertical axis was set to the effective dose calculated based on ICRP Publication 103 using Radimetrics. Radimetrics’ method for estimating the effective dose is based on Monte Carlo simulations. There are 54 different mathematical phantoms used for the estimation, which is based on the gender, age, and body size, and the phantom that most closely resembles the patient is selected. X-ray output information is collected from the Digital Imaging and Communication in Medicine Radiation Dose Structure Report (DICOM RDSR) and axial image header information [15]. Also, the SSDE was obtained from Radimetrics. To obtain the SSDE, Radimetrics first calculates the average CT value and the area of the ROI based on the axial image; SSDE is then derived automatically by Radimetrics by substituting the average CT values and ROI areas into the AAPM220 formula. The imaging area used in the Monte Carlo simulation is determined from the imaging area information in the DICOM tag or RDSR. The scan area is obtained from the DICOM RDSR. Then, the position is determined so that the center of the scan area corresponds to the center of the imaging site, and the organ doses and effective doses are calculated. According to the ICRP report, the use of effective doses is adapted for populations rather than for specific individuals. In this study, we took advantage of Radimetrics’ use of a wide variety of patient-specific mathematical phantoms to estimate the effective dose for each individual patient. Regarding the accuracy of Radimetrics estimates, Iriuchijima et al. concluded that the relative difference between measured and simulated organ doses is comparable to that of Fujii et al. [16] and is useful for assessing organ doses in individual patients [17]. The results of previous studies are similar to those reported in AAPM 246 [18], and the estimation accuracy of Radimetrics is considered comparable to that of existing software [16-18].

The slope of the single regression line was calculated from these data and was set as the k_SSDE-LP_. The SSDE in this study was the mean value obtained from each slice. The SSDE calculation method is described below.

$SSDE(z)=fD_{w}(z)\times CTDI_{vol}(z)$

where $fD_{w}(z)$ is the size-specific conversion factor in the AAPM report 204.

The method for calculating the average SSDE over the entire scan range is described.

$\overline{SSDE}=\frac{\sum_{Z=1}^{N}SSDE(z)}{N}$

where $N$ is the total number of images.

Similarly, k_DLP_ was determined from the slope of the single regression line with DLP plotted on the horizontal axis and effective dose plotted on the vertical axis.

When the effective dose estimated by DMS was positive, we verified the accuracy of the estimation by using the respective effective dose conversion coefficients (k_Conventional_, k_DLP_, and k_SSDE-LP_). We shall explain the specific verification methods. First, as noted above, we derived regression equations for k_DLP_ and k_SSDE-LP_ for males, females, and both sexes from the 2020 sample, which yielded six effective dose conversion coefficients. At this time, for k_Conventional_, the values for males, females, and both sexes were all set to 0.015 based on the ICRP report. The DLP and SSDE-LP were then multiplied by the applicable effective dose conversion factors for the 2021 sample. Specifically, the effective dose estimated by DMS was subtracted from the effective dose calculated, using each effective dose conversion factor. The effective dose conversion coefficients with the smallest discrepancies were then verified by calculating their means and standard deviations.

## Results

**Figure 1:**
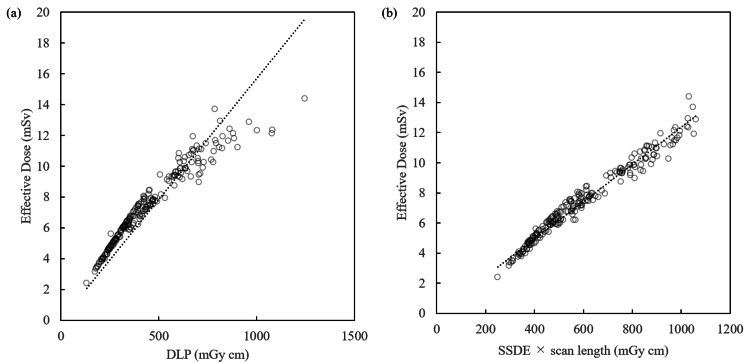
Regression equation for DLP and SSDE-LP, and effective dose (male) DLP: dose-length product; SSDE-LP: size-specific dose estimate

Figure 1a shows the scatter plots for males with the DLP plotted on the horizontal axis and effective dose on the vertical axis. Similarly, Figure 1b shows the scatter plots for males with the SSDE-LP values plotted on the horizontal axis and the effective dose plotted on the vertical axis. In Figure 1a, we see that when the DLP is lower or higher than the average, there is no plot on the line of the regression equation. When the same protocol is used, the DLP size reflects the patient’s body size. In other words, the calculation of the effective dose from the DLP yields a large difference from the calculated value for subjects deviating from the standard body shape. As can be seen from the scatter plots, the larger body size samples with larger DLP are more likely to deviate from the regression equation. On the other hand, Figure 1b shows that the differences from the calculated values are small over a wide range of body sizes.

**Figure 2:**
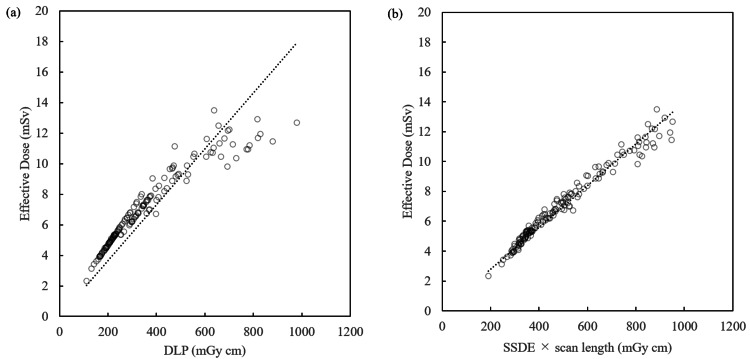
Regression equation for DLP, SSDE-LP, and effective dose (female) DLP: dose-length product; SSDE-LP: size-specific dose estimate

Figures 2a-2b show the same results for females. The slope is larger than that for the case of males. From this, it can be said that the effective dose can be estimated more precisely by subdividing the regression equation by sex.

**Figure 3:**
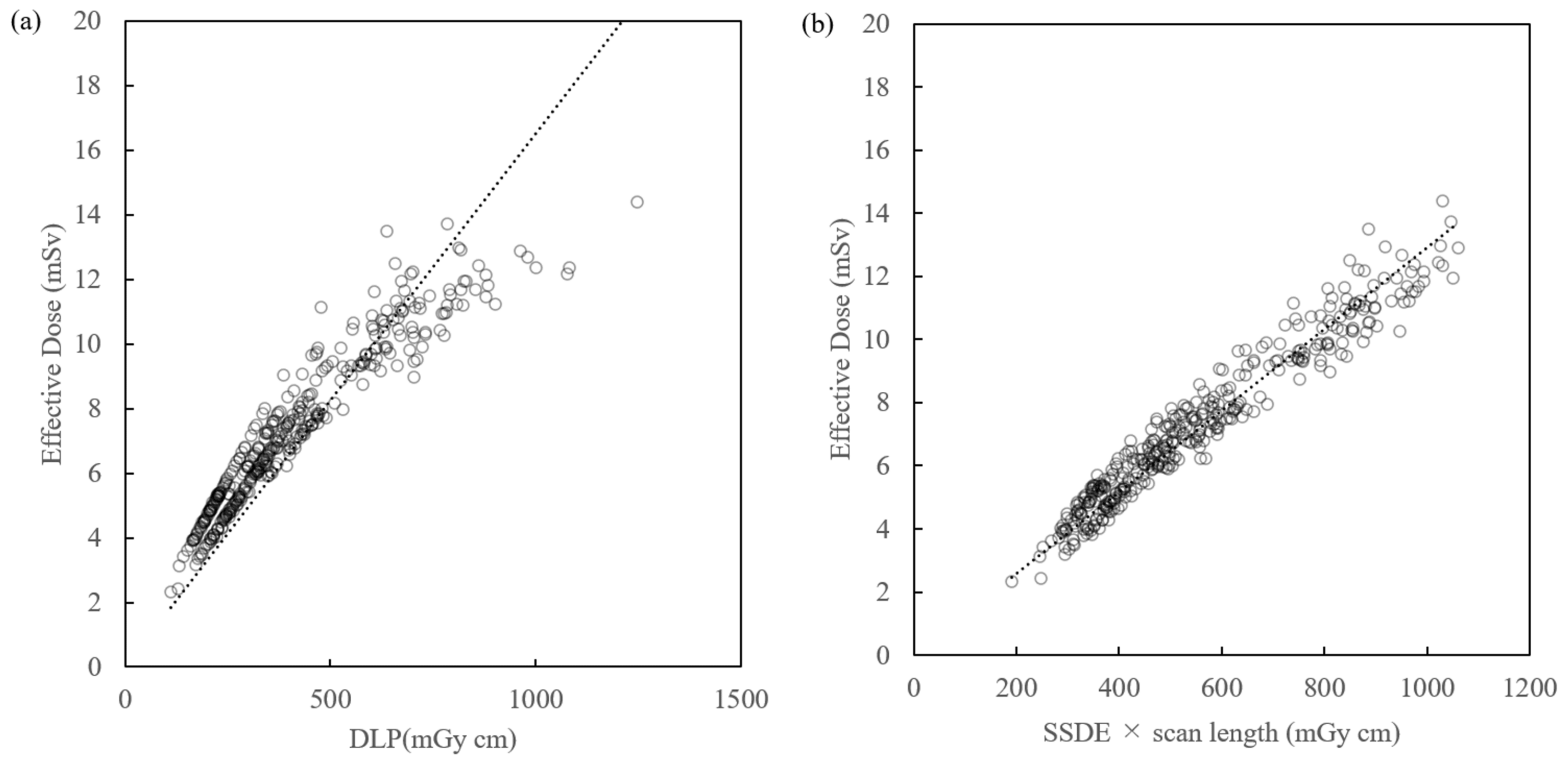
Regression equation for DLP, SSDE-LP, and effective dose (both genders) DLP: dose-length product; SSDE-LP: size-specific dose estimate

Figures 3a-3b are scatter plots including all samples, for both sexes. As expected, the slopes are between those in Figures 1 and 2. The k_DLP_ values for males, females, and both sexes were 0.016 (R^2^ = 0.981), 0.018 (R^2^ = 0.968), and 0.017 (R^2^ = 0.971), respectively (Figures 1-3, Table 3). The k_SSDE-LP_ values for males, females, and both sexes were 0.012 (R^2^ = 0.997), 0.014 (R^2^ = 0.996), and 0.013 (R^2^ = 0.993), respectively (Figures 1-3, Table 3). Thus, the R^2^ for k_SSDE-LP_ outperformed that for k_DLP_ in all cases.

**Table 3:** List of conversion factors calculated in this study

	Male	Female	Both genders	Target
k_Conventional_	0.015	0.015	0.015	DLP
k_DLP_	0.016	0.018	0.017	DLP
k_SSDE-LP_	0.012	0.014	0.013	SSDE-LP

To validate the accuracy of the regression equation, we quantitatively analyzed the deviations from the DMS estimates. Among the three effective dose conversion coefficients, k_SSDE-LP_ showed the smallest discrepancy for females and both sexes, followed by k_DLP_ and k_Conventional_ (Table 4). In males, both k_SSDE-LP_ and k_DLP_ showed only small discrepancies, followed by k_Conventional_. As for the standard deviation, that of k_SSDE-LP_ was the smallest for males, females, and both sexes.

**Table 4:** Range of divergence from DMS estimated effective dose when using each effective dose conversion factor DMS: Dose Management Software

	Male (mSv)	Female (mSv)	Both genders (mSv)
k_Conventional_	-0.7 ± 1.0	-1.8 ± 0.7	-1.2 ± 1.0
k_DLP_	-0.3 ± 1.2	-0.8 ± 1.0	-0.4 ± 1.4
k_SSDE-LP_	-0.3 ± 0.6	-0.7 ± 0.4	-0.2 ± 0.7

## Discussion

k_DLP_ is an effective dose conversion coefficient that improves the tissue weighting factor and mathematical phantom problems associated with k_Conventional_. As can be seen from the mean values of the discrepancies in Table 4, the k_DLP_ values were larger for males, females, and both sexes as compared to k_Conventional_. Therefore, we believe that using k_Conventional_ underestimates the effective dose.

The R^2^ for k_SSDE-LP_ was improved over that for k_DLP_, so that the former may be used to estimate the effective dose more accurately. Accuracy validation also showed the smallest discrepancies for males, females, and both sexes. Similarly, the standard deviations were also the smallest for all of males, females, and both sexes, which we believe ensures stable estimation accuracy. The usefulness of k_SSDE-LP_ is that it may allow the effective dose to be estimated with higher accuracy even for subjects with physiques deviating from the standard. The R^2^ was higher, and the accuracy of the effective dose estimation was improved by replacing CTDIvol with SSDE, a dosimetric index that takes information on the physique into account. The regression coefficients for each sex showed increased R^2^ values as compared with the case for both sexes, allowing more accurate estimation of the effective dose.

Large uncertainties regarding the estimation of the effective dose have been reported. However, as noted by Martin et al., the risk can be more appropriately assessed by combining it with the coefficients for the total lifetime risk of cancer development per unit effective dose tabulated by age at exposure and sex for the relevant CT examinations in the ICRP report [11]. From this perspective, it is important to estimate the effective dose as accurately as possible. The k_SSDE-LP_, which agrees well with the regression equation, has the highest estimation accuracy among the three and is highly significant in estimating effective dose.

There are some limitations of the k_SSDE-LP_ values derived in this study. Since a certain minimum number of samples is required to derive a reliable k_SSDE-LP_, it may be difficult to derive k_SSDE-LP_ values for imaging areas with a small number of examinations. It is necessary to verify if the same trend is observed for other imaging areas.

As mentioned earlier, we think that the accuracy of Radimetrics might be another limitation. However, the previous study has proven that there is no significant deviation from actual measurements as well as other previous methods, so we adopted Radimetrics for the present study [16].

In this article, we have reported a method using updated tissue weighting factors and mathematical phantoms, and incorporating the current technology of SSDE. However, since these may change over time, we believe that it is necessary to continue to update the data, as appropriate. In addition, as mentioned above, different combinations of DMS and CT equipment may provide different X-ray outputs and yield different effective dose estimates. Therefore, the effective dose conversion coefficients may also vary.

In addition, prior studies have noted limited information sharing with patients about the risk of radiation-induced cancer, despite growing concerns about medical radiation exposure [19]. It has also been reported that patients are unaware of the risk of radiation-induced cancer [20-22]. Therefore, it is important for physicians and other healthcare professionals to be aware of these risks and to share information with patients [23,24]. We believe that the application of the regression equations obtained in this study will help to provide appropriate patient explanations at other institutions.

## Conclusions

The concept represented by k_Conventional_, which allows the estimated effective dose to be quickly calculated from the DLP displayed on the device console, does not require dedicated software and is in high demand in busy clinical practice. However, k_Conventional_, which has been used in some recent studies, has some problems, as described earlier in the text. We derived a new effective dose conversion coefficient, k_SSDE-LP_, from a simple regression equation derived by plotting SSDE-LP and the effective dose. In addition, a different sample from that used to derive the regression equation was used to verify that the effective dose could be estimated with a high degree of accuracy. Our results revealed that k_SSDE-LP_ can be used to estimate the effective dose more accurately than the conventionally used conversion coefficients.

In this study, conversion factors were derived for the abdominopelvic region in adults. As a future perspective, we would like to derive conversion coefficients for the thoracic region and children, as various protocols are used in clinical practice depending on the region and age.
